# Psychometric Properties of the Brazilian Version of the Sport Anxiety Scale-2

**DOI:** 10.3389/fpsyg.2019.00806

**Published:** 2019-04-16

**Authors:** Viviane Vedovato Silva-Rocha, Diogo Araújo de Sousa, Flávia L. Osório

**Affiliations:** ^1^Department of Neuroscience and Behavioral Sciences, Ribeirão Preto Medical School, University of São Paulo, Ribeirão Preto, Brazil; ^2^Department of Psychology, Pio Tenth College, Aracaju, Brazil; ^3^Department of Neurosciences and Behavioral Sciences, Ribeirão Preto Medical School, University of São Paulo, Ribeirão Preto, Brazil

**Keywords:** performance anxiety, competitive anxiety, cross-cultural adaptation, psychometrics, reliability, validity

## Abstract

Competitive anxiety (CA) is an emotional reaction manifested at a somatic and/or cognitive level that regularly appears before or during sports competitions and can significantly impact an athlete’s performance. Given the scarcity of validated instruments available for evaluating the competitive-anxiety trait in the Brazilian context, this study aimed to investigate the psychometric properties of the Sport Anxiety Scale-2 (SAS-2). The study sample was composed of 238 professional and amateur athletes aged 13 years or older who practice different sports modalities. The results of confirmatory factor analysis (CFA) revealed adequate fit indices of the original three-factor theoretical model of the SAS-2 after including a correlation between the errors for items 6 and 12 of the somatic anxiety subscale (CFI = 0.97, TLI = 0.96, RMSEA = 0.08, WRMR = 1.04). For convergent and divergent validity, the SAS-2 subscales exhibited a positive and strong correlations with the Competitive State Anxiety Inventory-2R (CSAI-2R; *r* = 0.52–0.82), weak to moderate correlations with the State-Trait Anxiety Inventory – only the trait scale (STAI-T; *r* = 0.49–0.59), weak correlations with the Social Phobia Inventory (SPIN; *r* = 0.29–0.41) and weak to moderate correlations with the Patient Health Questionnaire (PHQ-9; *r* = 0.49–0.56). The SAS-2 was also able to discriminate among participants with and without social anxiety, general trait anxiety and depressive symptoms, thus confirming its discriminant validity. According to ROC curve analysis, the cutoff point at a score of 29 indicated the optimal balance of sensitivity (0.74) and specificity (0.82). The internal consistency (α = 0.73–0.86) and the test-retest reliability (ICC = 0.73–0.80) were satisfactory. These results indicated that the Brazilian version of the SAS-2 exhibited satisfactory psychometric performance and could be used in the Brazilian context.

## Introduction

Anxiety has been considered an emotional response necessary for performing certain tasks; however, depending on its intensity and duration and its negative impact and psychological suffering for an individual, it can be considered pathological (Steimer, 2002; Brandão, 2005; Marques et al., 2016; Khan et al., 2017). Among anxiety disorders, performance anxiety is classified as a subtype of social anxiety disorder (SAD) – intense anxiety or fear of being judged, negatively evaluated, or rejected in a social or performance situation – and is characterized by marked fear of speaking or performing in public; it mainly affects individuals involved in performance contexts, such as musicians, dancers, artists and athletes (Associação Americana de Psiquiatria [APA], 2014).

Regarding the sporting context, competition is considered a potentially anxiogenic situation, since public exposure involves scrutiny by others and the association of an athlete’s image with his or her performance. Moreover, anxiety tends to be present even in cases of good performance history, since the end result is always uncertain (Frischnecht, 1990; Martens et al., 1990). The impact of anxiety on athletes’ lives has been studied for many years, and the results indicate that high levels of anxiety are inversely associated with sports performance (Milavić et al., 2013; Mottaghi et al., 2013; Judge et al., 2016), since anxiety can cause physiological (energy expenditure and cardiovascular changes), motor (impaired coordination), cognitive (reduced attention, concentration, and decision-making capacity) and relational (increased conflict among team members) changes (Guzmán et al., 1995; Mesquita and Todt, 2000).

Considering the particularities of performance anxiety in sports, the term competitive anxiety (CA) was coined to refer to the emotional reaction expressed at the somatic and/or cognitive level that appears regularly before or during sports competitions (Martens et al., 1990). The somatic dimension of CA involves different physiological reactions, such as muscle tension, tachycardia, flushing, tremors, and sweating. The cognitive dimension includes the content of thoughts, such as self-preoccupation, poor performance, negative evaluation, social comparison, expectations, and demands from the coaching staff, team, family, and crowd (Guzmán et al., 1995; De Rose Júnior et al., 2004; Wallhead and Ntoumanis, 2004; Vieira et al., 2011).

In addition to its somatic and cognitive dimensions, anxiety can also be classified according to its state-trait typology. The first type of anxiety is experienced transiently, whereas the second is considered a relatively stable tendency of individuals to perceive different situations of daily life as threatening (Spielberger et al., 1983). The most common multidimensional scales with signs and symptoms used for assessing competitive state anxiety experienced by athletes are the CSAI-2 (Martens et al., 1990) and CSAI-2R (Cox et al., 2003), whereas the most common scale for assessing competitive trait anxiety is the SAS-2 (Smith et al., 2006). This last one is the focus of this study because the previous instruments have already been examined in psychometric studies in the Brazilian context.

The SAS-2 was originally developed in English as a multidimensional instrument composed of 15 items equally distributed across three subscales (somatic anxiety: items 2, 6, 10, 12, and 14; worry: items 3, 5, 8, 9, and 11; concentration disruption: items 1, 4, 7, 13, and 15) and scored on a 4-point scale of intensity (1 = not at all to 4 = very much). The score is obtained by summing the items of each subscale and ranges from 5 to 20 points, where the highest score indicates a high probability of CA (Smith et al., 2006).

The psychometric qualities of this instrument were originally measured in samples of children (9 to 14 years old, *n* = 1038) and adults (university students, *n* = 1294) of both sexes who played various sports (basketball, volleyball, soccer, and hockey). CFA indicated that the three-factor structure was the most appropriate [comparative fit index (CFI) = 0.95–0.97; non-normed fit index (NNFI) = 0.95–0.96; root mean square error of approximation (RMSEA) = 0.05–0.065], and satisfactory values were also found for internal consistency (α ≥ 0.84) and test-retest reliability (≥0.76) (Smith et al., 2006).

Subsequently, the SAS-2 was adapted and studied from a psychometric perspective for different languages/countries, such as Spain (Ramis et al., 2010), Belgium (Jannes et al., 2011), and Portugal (Sousa et al., 2011). The study by Ramis et al. (2015), with a sample of 842 athletes from these three countries, confirmed the aforementioned factorial structure for all versions of the SAS-2, suggesting that the instrument can be used in research regardless of the language, gender, age, and type of sports played by the participants.

In Brazil, the cross-cultural adaptation of the SAS-2 was conducted by Rocha and Osório (2018), demonstrating satisfactory content validity. Following the previous research, the current study aimed to investigate the psychometric properties of construct validity (CFA and convergent, divergent, and discriminant validity) and reliability (internal consistency and test-retest reliability) of this scale in the Brazilian context.

## Materials and Methods

This study was approved by the local ethics committee (process HCRP no. 17533/2015) and conducted according to Resolution 466 of 2012 of the Brazilian National Health Council for research with human subjects (Ministério da Saúde and Conselho Nacional de Saúde, 2012). All subjects voluntarily participated in the study and signed the informed consent form.

The following inclusion criteria were adopted to select the sample: amateur and professional athletes of any of a variety of sports modalities and of either sex, age ≥13 years, and participation in sports competitions at least once per year. Of the 333 athletes contacted, 95 were excluded because they did not agree to participate in the study, were absent from the training site when the study was presented/explained (in the case of live data collection) or did not return the research protocol at the agreed-upon time. Thus, the final sample was composed of 238 subjects.

In addition to the Brazilian version of the SAS-2 (Rocha and Osório, 2018), the following instruments were used for the study to examine the convergent, divergent and discriminant validity:

### Competitive State Anxiety Inventory-2R (CSAI-2R)

In this short version of the CSAI-2, the objective is to evaluate state anxiety in athletes during sports competitions. This self-administered instrument is composed of 17 items distributed across three subscales (the somatic anxiety subscale includes 7 items, the cognitive anxiety subscale includes 5 items, and the self-confidence subscale includes 5 items), scored on a four-point Likert scale (1 = not at all to 4 = very much). The score of each subscale is calculated by summing the respective items divided by the number of items, ranging from 1 to 4 points (Cox et al., 2003). An adapted version with demonstrated validity for the Brazilian context was used (α > 0.70; CFI = 0.96, GFI = 0.94, RMSEA = 0.044) (Coelho et al., 2010; Fernandes et al., 2012).

### Social Phobia Inventory (SPIN)

This self-administered instrument was developed to evaluate the presence of social anxiety indicators, and it consists of 17 items scored on a five-point Likert scale (0 = not at all to 4 = extremely) distributed across three subscales (the fear subscale includes six items, the avoidance subscale includes seven items, and the physiologic arousal subscale includes four items), with total scores ranging from 0 to 68 points (Connor et al., 2000). An adapted version with demonstrated validity for the Brazilian context was used (total scale: α = 0.90, fear subscale: α = 0.80, avoidance subscale: α = 0.78, physiologic arousal: α = 0.71; sensitivity = 0.84, and specificity = 0.87) (Osório et al., 2008).

### State-Trait Anxiety Inventory (STAI – Trait Scale)

This self-administered instrument was developed to evaluate trait anxiety, and it consists of 20 items scored on a four-point Likert scale (1 = almost never to 4 = almost always) (Spielberger et al., 1983). An adapted version with demonstrated validity for the Brazilian context was used (α = 0.88) (Biaggio and Natalício, 1979; Fioravanti et al., 2006).

### Patient Health Questionnaire (PHQ-9)

This self-administered instrument was developed to evaluate the presence of depressive symptoms, and it consists of nine items scored on a four-point Likert scale (0 = not at all to 3 = nearly every day) (Spitzer et al., 1994). An adapted version with demonstrated validity for the proposed Brazilian context was used (cutoff score = 10, sensitivity = 1.00, specificity = 0.98, positive predictive value = 0.97, and negative predictive value = 1.00) (Osório et al., 2009).

For the data collection, the subjects were contacted at the training centers or over the internet, and those who agreed to participate received a notebook with the instruments described above for self-administration. The average time spent answering the instrument was 50 min, and the researcher was available to answer any questions. For the study of test-retest reliability, part of the sample (*n* = 50) was randomly selected to fill out the SAS-2 seven to 15 days after the first administration.

The data were manually coded and inputted into databases using the IBM Statistical Package for the Social Sciences (SPSS) version 23.0 for descriptive and inferential analysis and Mplus software version 7.0 for factor analysis. Descriptive statistical analyses were performed to characterize the sample. Construct validity was analyzed using CFA, in addition to convergent, divergent and discriminant validity (known groups).

The following parameters were used for the fit indices in the CFA: CFI (acceptable ≥ 0.90; good ≥ 0.95), Tucker-Lewis index – TLI (acceptable ≥ 0.90; good ≥ 0.95), RMSEA (acceptable ≤ 0.08; good ≤ 0.05), and weighted root mean square residual – WRMR (good ≤ 1.00). The RMSEA was calculated with a 90% confidence interval. Standardized regression weights (i.e., factor loadings) were calculated for the items in each of the scale factors, with scores ≥0.40 considered to be adequate (Hu and Bentler, 1999; Brown, 2006; Muthén and Muthén, 2012).

The Pearson correlation coefficient was used for the analysis of convergent/divergent validity, and the values were interpreted according to the following parameters: irrelevant (*r* = 0–0.30), weak (*r* = 0.30–0.50), moderate (*r* = 0.50–0.70), strong (*r* = 0.70–0.90), and very strong (*r* = 0.90–1.00) (Hinkle et al., 2003). Student’s *t*-test was used in the analysis of discriminant validity for comparison of the following known groups: (a) With and without social anxiety, adopting the cutoff score ≥19 on the SPIN (Osório et al., 2008); (b) with and without trait anxiety, adopting as a cutoff the mean score of ≥40.3(♂) and ≥44.7(♀) on the STAI (Fioravanti et al., 2006); and (c) with and without depressive symptoms, with a cutoff score ≥10 on the PHQ-9 (Osório et al., 2009). Moreover, receiver operating characteristic (ROC) analysis was used to determine the optimal cutoff point (OCP) of the SAS-2. To do so, Youden’s J index – the smallest sum of the classification error rates – was calculated from the sum of sensitivity and specificity minus one (i.e., J = sensitivity + specificity − 1) (Böhning et al., 2008).

Reliability was assessed by internal consistency, i.e., calculating Cronbach’s alpha, with values ≥0.70 considered satisfactory (Hair et al., 2009). Test-retest reliability was based on the intraclass correlation coefficient (ICC), which was interpreted according to the following parameters: poor (0–0.20), reasonable (0.21–0.40), good (0.41–0.60), very good (0.61–0.80), and excellent (0.81–1.00) (Weir, 2005). The correlation between the SAS-2 items was also evaluated, with item-total correlations ≥0.50 and interitem correlations ≥0.30 considered satisfactory (Hair et al., 2009).

A level of significance of *p* ≤ 0.05 was adopted for all psychometric analyses.

## Results

### Sample Characteristics

The sample of 238 Brazilian athletes was predominantly composed of male (♂ = 169; ♀ = 69), adult (13–18 years = 77; 19–53 years = 161; *X* = 22.9 ± 7.9), and single (without partner = 197; with partner = 41) subjects who had achieved a higher level of education (up to 12 years of study = 113; over 12 years of study = 125) and who were engaged in other occupational activities in addition to their sport (athlete = 108; another profession = 130).

More than half of the athletes practiced collective sports (*n* = 127); had high performance (*n* = 132); had up to 9 years of experience in sports (*n* = 127); practiced only one sport (*n* = 160); trained at least three times per week (*n* = 225); and participated in up to three championships/competitions per year (*n* = 124) at the municipal/regional (*n* = 36), state/national (*n* = 171), and international (*n* = 31) levels. The sports practiced included soccer, swimming, volleyball, judo, and track and field.

Regarding mental health indicators, Table 1 draws attention to the significant prevalence of social anxiety symptoms, depression, and alcohol abuse among the athletes.

**Table 1:** Mental health indicators for the sample (*n* = 238).

Psychiatric indicators	Evaluation instruments	*X*(*SD*)	Cutoff score	Prevalence *n* (%)
Trait anxiety	STAI-T	38.5(8.8)	–	–
Social anxiety	SPIN	10.7(7.9)	≥19	35(14.7)
Depression	PHQ-9	4.9(4.4)	≥10	34(14.3)
Alcohol abuse	FAST	1.5(2.1)	≥3	54(22.7)

*SD, standard deviation; FAST, fast alcohol screening test; STAI-T, State-Trait Anxiety Inventory (trait scale); n, number of subjects; PHQ-9, patient health questionnaire; SPIN, social phobia inventory; %, percentage.*

### Confirmatory Factor Analysis

The original SAS-2 model (Smith et al., 2006) was evaluated using CFA, and the results are shown in Table 2.

**Table 2:** Fit indices obtained in the confirmatory factor analysis (CAF).

MODEL	χ^2^ / df / *p*	CFI	TLI	RMSEA	WRMR
Model 1^a^	412.233 / 87 / < 0.001	0.91	0.90	0.125	1.550
Model 2^xxb^	215.713 / 86 / < 0.001	0.97	0.96	0.080	1.042

*^a^Original model (Smith et al., 2006); ^b^original model with added correlation between the errors of items 6 and 12; χ^2^, chi-square; CFI, comparative fit index; df, degrees of freedom; RMSEA, root mean square error of approximation; TLI, tucker-lewis index; WRMR, weighted root mean square residual.*

**FIGURE 1:**
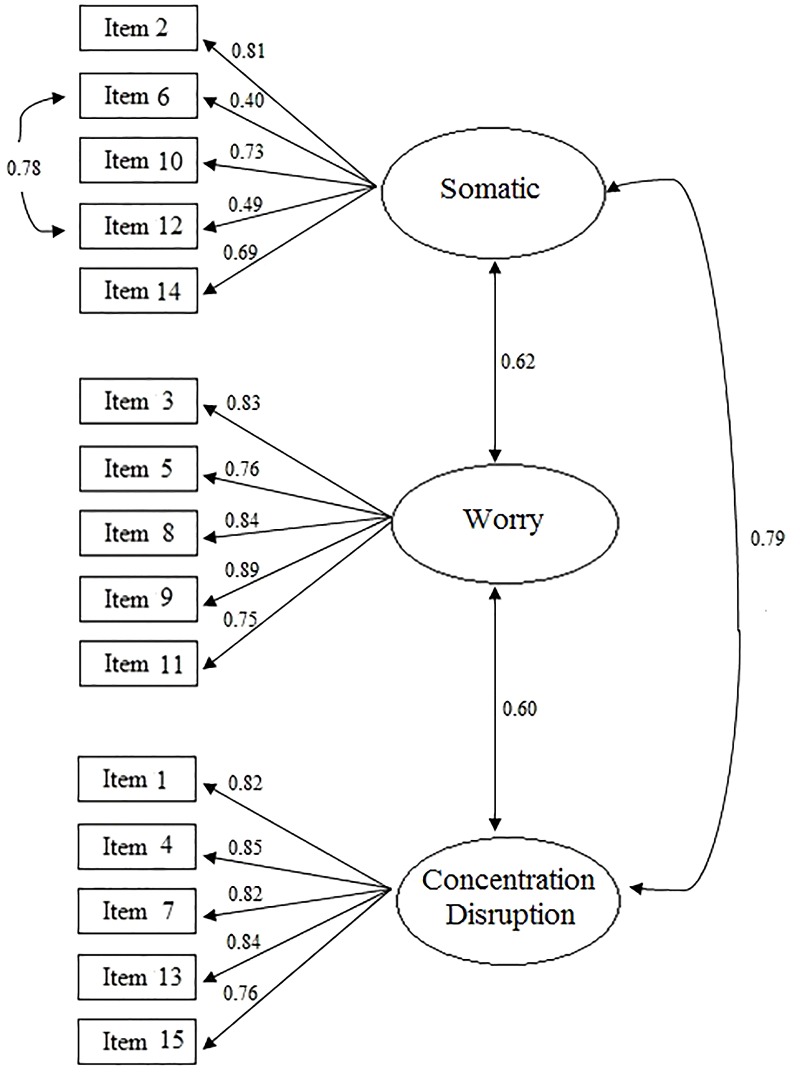
Path diagram of Model 2 of the SAS-2.

Regarding the fit to the data from the Brazilian sample, the fit indices for the original SAS-2 model (Model 1) were not good (CFI and TLI) or were unacceptable (RMSEA). The analysis of the modification indices suggested in the CFA identified that the model fit could be substantially improved by including a correlation between the errors of items 6 (“I feel tense in my stomach”) and 12 (“My stomach feels upset”) on the somatic anxiety subscale. Given this, a new CFA was conducted (Model 2) to test the original scale model with the addition of the correlation between the errors of these items, and the results showed a considerable improvement in fit, with good CFI and TLI values and acceptable RMSEA and WRMR values. Figure 1 shows the path diagram of Model 2 according to the CFA.

Figure 1 shows that all items presented satisfactory factor loadings (that is, equal to or greater than 0.40), as well as moderate to strong correlations between the three factors, with values ranging from 0.60 to 0.79. Thus, the original three-factor model, with the added correlation between items 6 and 12, can be considered appropriate for the Brazilian context.

### Item Analysis

The means of the raw scores of each SAS-2 subscale were calculated, with higher levels of cognitive anxiety symptoms manifesting as worry (worry subscale = 11.87 ± 3.53; somatic anxiety subscale = 8.50 ± 2.37; concentration disruption subscale = 7.34 ± 2.41).

The item-total correlation coefficients were satisfactory except for the somatic anxiety subscale, in which items 2, 6, and 10 presented values lower than 0.50. This same subscale was also the only one to present item-item correlation coefficients below the expected value of 0.30 (see Supplementary Table S1).

### Convergent/Divergent Validity

To examine convergent/divergent validity, the SAS-2 factorial score was used, and the results are presented in Table 3.

**Table 3:** Convergent/divergent validity indicators of the SAS-2.

Scale	1	2	3	4	5	6	7	8	9
(1) SAS-2 – SOM	1	0.71^∗^	0.89^∗^	0.77^∗^	0.64^∗^	−0.35^∗^	0.41^∗^	0.57^∗^	0.49^∗^
(2) SAS-2 – WO		1	0.67^∗^	0.52^∗^	0.82^∗^	−0.35^∗^	0.29^∗^	0.49^∗^	0.49^∗^
(3) SAS-2 – CD			1	0.61^∗^	0.62^∗^	−0.43^∗^	0.36^∗^	0.59^∗^	0.56^∗^
(4) CSAI-2R – SOM				1	0.56^∗^	−0.20^∗^	0.47^∗^	0.51^∗^	0.41^∗^
(5) CSAI-2R – COG					1	−0.41^∗^	0.36^∗^	0.48^∗^	0.43^∗^
(6) CSAI-2R – SC						1	−0.17^∗^	−0.42^∗^	−0.29^∗^
(7) SPIN							1	0.48^∗^	0.27^∗^
(8) STAI – Trait								1	0.63^∗^
(9) PHQ-9									1

*^∗^p ≤ 0.05; CD, concentration disruption; COG, cognitive anxiety; SC, self-confidence; SOM, somatic anxiety; WO, worry.*

As expected, the correlations between the SAS-2 subscales were positive and ranged from moderate to strong in magnitude. The SAS-2 subscales of somatic anxiety and worry were positively and strongly correlated with the somatic and cognitive anxiety subscales of the CSAI-2R, respectively. The SAS-2 concentration disruption subscale showed a moderate positive correlation with the somatic and cognitive anxiety subscales of the CSAI-2R, attesting to its convergent validity.

Considering the correlated constructs, the SAS-2 subscales showed a positive but weak correlation with the SPIN and a weak to moderate correlation with the STAI-T. Regarding divergent validity, the three subscales of the SAS-2 were negatively and weakly correlated with the CSAI-2R self-confidence subscale, whereas the correlation with PHQ-9 was positive and weak (somatic anxiety and worry) to moderate (concentration disruption).

### Discriminant Validity

The results showed that the overall score of the SAS-2 and the subscale scores could discriminate among participants regarding the presence of social anxiety, trait anxiety and depressive symptoms (see Supplementary Table S2).

**FIGURE 2:**
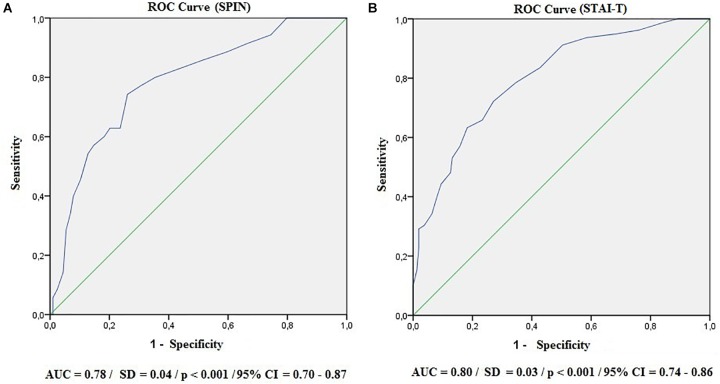
Receiver operating characteristic curves (ROC). AUC, area under the curve; SD, standard deviation; CI, confidence interval. **(A)** SPIN; **(B)** STAI-T.

Subsequently, ROC curve analysis was performed to identify the discriminant capacity of the SAS-2 with the SPIN and STAI-T scales used as parameters. The curves are shown in Figure 2.

With reference to the SPIN (A) and STAI-T (B) instruments, the area under the curve presented satisfactory values (AUC > 0.70). To find the ideal cutoff point for SAS-2, the sensitivity, specificity and Youden’s J indices were used. The results are shown in Table 4.

**Table 4:** Sensitivity and specificity indicators and Youden’s J index for the different SAS-2 cutoff points using the SPIN and STAI-T instruments as references.

	SPIN			STAI-T		
Score	Sensitivity	Specificity	J Index	Sensitivity	Specificity	J Index
>22	0.94	0.26	0.20	0.95	0.31	0.26
>23	0.91	0.34	0.25	0.94	0.42	0.35
>24	0.89	0.40	0.29	0.91	0.50	0.41
>25	0.86	0.49	0.35	0.84	0.57	0.41
>26	0.83	0.57	0.40	0.79	0.65	0.44
>27	0.80	0.65	0.45	0.72	0.73	0.45^∗^
>28	0.77	0.70	0.47	0.66	0.77	0.43
>29	0.74	0.74	0.48^∗^	0.63	0.82	0.45^∗^
>30	0.63	0.76	0.39	0.57	0.84	0.41
>31	0.63	0.80	0.43	0.53	0.87	0.40
>32	0.60	0.82	0.42	0.48	0.87	0.36
>33	0.57	0.85	0.42	0.44	0.91	0.35
>34	0.54	0.87	0.42	0.41	0.92	0.32
>35	0.46	0.90	0.35	0.34	0.94	0.28
>36	0.40	0.92	0.32	0.30	0.96	0.27
>37	0.34	0.93	0.27	0.29	0.98	0.27

*^∗^Highest value found for the J index.*

According to the highest value found for the J index (SPIN = 0.48; STAI-T = 0.45), a score of 29 is the ideal cutoff because it better balances the sensitivity (SPIN = 0.74; STAI-T = 0.63) and specificity (SPIN = 0.74; STAI-T = 0.82) values for the two parameters used. Notably, certain cutoff scores maximize sensitivity without reducing specificity to less than 50% and vice versa, thus suggesting a score of 26 as an appropriate cutoff to favor sensitivity (SPIN = 0.83; STAI-T = 0.79) and a score of 31 to favor specificity (SPIN = 0.80; STAI-T = 0.87).

### Reliability

The SAS-2 presented adequate internal consistency, since Cronbach’s alpha was satisfactory for the three subscales (somatic anxiety = 0.73; worry = 0.86; concentration disruption = 0.83; total scale = 0.88) and since no item, if excluded, significantly affected the alpha value and the variance (see Supplementary Table S3).

Similarly, the test-retest reliability indicators were very good for the total scale and for the subscales [somatic anxiety: ICC = 0.80 (95% CI = 0.66–0.88); worry: ICC = 0.74 (95% CI = 0.57–0.85); concentration disruption: ICC = 0.73 (95% = 0.58–0.84); total scale: ICC = 0.80 (95% CI = 0.66–0.89)].

## Discussion

The present study investigated the psychometric properties of the Brazilian version of the SAS-2, and its validity and reliability indicators were analyzed using different techniques.

The instrument’s structure was assessed by CFA, which indicated that the original three-factor model of the SAS-2 initially did not fit well with the Brazilian data, and the addition of a correlation between the errors of items 6 (“I feel tense in my stomach”) and 12 (“My stomach feels upset”) of the somatic anxiety subscale was suggested. Thus, a new CFA was conducted by including the correlation between the errors of these items, and the results yielded satisfactory indices and were in agreement with the study of the original version of the scale (CFI = 0.95 to 0.97; NNFI = 0.94 to 0.97; RMSEA = 0.04 to 0.07) (Smith et al., 2006) and the Spanish version (CFI = 0.98; TLI = 0.99; RMSEA = 0.05) (Ramis et al., 2010).

Considering that the items have different factorial loadings and that the errors of items 6 and 12 are correlated in this particular model, it is worth noting that the factorial score calculated for each of the SAS-2 factors is, for research purposes, more adequate than the use of the raw score calculated from the simple sum of the items in each subscale (see the syntax for calculating the factorial score in Supplementary Table S4).

The adjusted model was chosen because of the possibility of comparing scores between samples from Brazil and other countries that use the SAS-2 with the original three-factor model. However, in clinical or technical-professional practice, with limited statistical apparatuses, calculating the raw score for each SAS-2 factor is useful as an approximate reference for the results presented in this study using factorial scores.

Another observation to be highlighted is that evaluation with this scale may require qualitative consideration with respect to items 6 and 12, since the terms “tense in my stomach” and “upset stomach” could have been understood as the same symptom, therefore indicating a need for future revision.

For examining convergent validity, we followed the recommendation in the literature to use instruments that evaluate correlated constructs in the absence of a gold standard (Souza et al., 2017). Determining which instruments to use in examining convergent and divergent validity tends to be challenging for the field of psychometry because it is not always possible to choose the optimal method because of the shortage of instruments considered to be the gold standard.

The original study used the first version of the SAS and instruments that assess other constructs, such as achievement goal orientations, motivational climate, self-esteem, social desirability and perceived competence for this type of analysis (Smith et al., 2006). In Portugal, only the constructs of achievement goal orientations and motivational climate were used (Sousa et al., 2011), whereas the Belgian study focused on personality hierarchy and state-trait anxiety (Jannes et al., 2011). The Spanish version, by contrast, was not subjected to the study of convergent and divergent validity (Ramis et al., 2010).

The present study in turn based its choice on instruments widely used in national and international studies and validated in the Brazilian context, considering them to be the most adequate for the proposed objectives (Sousa et al., 2013; Barros et al., 2017). The somatic and cognitive anxiety subscales of the CSAI-2R were thus selected to represent the construct CA and trait scale of the STAI and SPIN in order to represent the constructs of general anxiety and social anxiety, respectively, with the hypothesis of at least moderate correlations.

The results indicate the presence of significant correlations that became stronger as the proximity of the constructs improved. The correlations between the SAS-2 worry and the CSAI-2R cognitive anxiety subscales as well as between the SAS-2 and CSAI-2R somatic anxiety subscales were notable, as they were strong (*r* ≥ 0.77).

The SAS-2 subscales were moderately correlated with the STAI-I and weakly correlated with the SPIN, signaling the specificity of the CA construct and the importance of developing specific instruments to assess anxiety in the sports context.

The CSAI-2R self-confidence subscale and the PHQ-9 were used to study divergent validity. Thus, it was found that the SAS-2 was weakly and inversely correlated with self-confidence, attesting to its divergence from this construct. In relation to the PHQ-9, the correlation values varied from weak to moderate, pointing to divergence between the constructs but signaling the comorbidity prevalent between them in the clinical context.

This pioneering analysis of the discriminant validity of the SAS-2 used correlated constructs as a reference, given the absence of a gold standard for evaluating CA, as previously mentioned. The results show that the Brazilian version was able to distinguish groups with and without psychopathological indicators (general trait anxiety, social anxiety, and depression). The sensitivity and specificity of the instrument were analyzed from the ROC curve, suggesting that a score of 29 was the most appropriate for the screening of individuals with pathological levels of CA. This cutoff score has favorable levels of both sensitivity and specificity (≥63%); however, other close cutoff scores are able to maximize these indicators without significantly increasing the false positive and negative rates. This is especially important when the instrument is used for screening.

In addition to indicators of validity, indicators of reliability were also excellent according to the parameters established by Hair et al. (2009). The internal consistency reached values close to those of the original study, in which alpha ranged from 0.84 to 0.91. The temporal stability was tested over a period of 7 to 15 days, with acceptable values that were also very close to those of the original study (Smith et al., 2006). Notably, unlike the original study, which examined test-retest reliability only with skaters, the present study used a sample of athletes that was broader and more diverse, especially with regard to sports modalities.

Thus, this psychometric study of the SAS-2 not only revealed its suitability for use in the Brazilian context but also found new evidence of validity and reliability hitherto not explored in previous studies with different versions of the instrument. Thus, the scale is available and can freely be used in clinical and research contexts. The availability of screening instruments such as the SAS-2 can facilitate the identification of groups with potential risk and guide the planning of more effective interventions to support athletes’ performance and quality of life.

However, the study was not without limitations: (1) The sample size was not large; (2) the sample contained a significant number of amateur athletes; and (3) the study did not use a gold standard (external criterion) to analyze the discriminant validity and establish the cutoff score for the studied scale. It is recommended that future studies consider the possibility of evaluating only high-performance athletes, since performance anxiety may have different meanings for a professional athlete and an amateur. Future research could also use more refined instruments for the diagnosis of psychiatric disorders such as generalized anxiety, social anxiety and depression, notably the Structured Clinical Interview for Diagnostic and Statistical Manual of Mental Disorders (SCID – DSM-V) (Associação Americana de Psiquiatria [APA], 2014), or even a clinical evaluation, in order to contribute to the enrichment and robustness of the data.

## Ethics Statement

This study was approved by the local ethics committee (process HCRP No. 17533/2015) and conducted according to Resolution 466 of 2012 of the Brazilian National Health Council for research with humans. All subjects voluntarily participated in the study and signed the informed consent form.

## Author Contributions

FLO and VS-R conceived and designed the work. VS-R collected the data. FLO, DdS, and VS-R analyzed and interpreted the data. VS-R drafted the manuscript. FLO and DdS critically revised the manuscript. FLO approved the final version of the manuscript.

## Conflict of Interest Statement

The authors declare that the research was conducted in the absence of any commercial or financial relationships that could be construed as a potential conflict of interest.
