# Supplementary material for: Psychometric Properties of the Brazilian Version of the Sport Anxiety Scale-2
Source: Front Psychol. 2019 Apr 16;10:806. doi: 10.3389/fpsyg.2019.00806 (PMC6477035; doi:10.3389/fpsyg.2019.00806)
Supplement: Supplementary file 1 [file Table_1.docx]

**Supplementary Table S1.** Item-total and item-item correlation matrix of the SAS-2 subscales.

| **Subscale** | **Items** | **Item-total correlation** | **Item-item correlation** | | | | |
| --- | --- | --- | --- | --- | --- | --- | --- |
|  |  |  | **2** | **6** | **10** | **12** | **14** |
| Somatic | 2  6  10  12  14 | 0.46  0.48  0.46  0.60  0.50 | 1 | 0.27  1 | 0.36  0.24  1 | 0.28  0.69  0.28  1 | 0.43  0.20  0.46  0.38  1 |
| **Subscale** | **Items** | **Item-total correlation** | **3** | **5** | **8** | **9** | **11** |
| Worry | 3  5  8  9  11 | 0.70  0.65  0.73  0.79  0.52 | 1 | 0.60  1 | 0.61  0.53  1 | 0.63  0.61  0.74  1 | 0.41  0.37  0.46  0.51  1 |
| **Subscale** | **Items** | **Item-total correlation** | **1** | **4** | **7** | **13** | **15** |
| Concentration disruption | 1  4  7  13  15 | 0.63  0.69  0.65  0.64  0.58 | 1 | 0.58  1 | 0.54  0.56  1 | 0.48  0.51  0.51  1 | 0.40  0.49  0.44  0.53  1 |
