# Supplementary material for: Psychometric Properties of the Brazilian Version of the Sport Anxiety Scale-2
Source: Front Psychol. 2019 Apr 16;10:806. doi: 10.3389/fpsyg.2019.00806 (PMC6477035; doi:10.3389/fpsyg.2019.00806)
Supplement: Supplementary file 2 [file Table_2.docx]

**Supplementary Table S2.** Discriminant validity (known groups) of the SAS-2 subscales.

| **Known groups** | ***n*** | **Somatic**  **X(SD)** | **Worry**  **X(SD)** | **Concentration disruption**  **X(SD)** | **Total**  **X(SD)** |
| --- | --- | --- | --- | --- | --- |
| With social anxiety | 35 | 10.31(2.81) | 14.29(2.89) | 9.03(2.67) | 33.63(6.29) |
| Without social anxiety | 203 | 8.19(2.14) | 11.46(3.47) | 7.05(2.24) | 26.69(6.41) |
| Statistic |  | *p* ≤ 0.01 | *p* ≤ 0.01 | *p* ≤ 0.01 | *p* ≤ 0.01 |
|  |  |  |  |  |  |
| With trait anxiety | 79 | 9.75(2.64) | 13.84(3.46) | 9.04(2.61) | 32.62(6.85) |
| Without trait anxiety | 159 | 7.88(1.95) | 10.90(3.16) | 6.50(1.78) | 25.28(5.38) |
| Statistic |  | *p* ≤ 0.01 | *p* ≤ 0.01 | *p* ≤ 0.01 | *p* ≤ 0.01 |
|  |  |  |  |  |  |
| With depressive symptoms | 34 | 10.03(3.29) | 14.74(3.64) | 9.76(2.85) | 34.53(7.44) |
| Without depressive symptoms | 204 | 8.25(2.10) | 11.40(3.29) | 6.94(2.07) | 26.58(6.04) |
| Statistic |  | *p* ≤ 0.05 | *p* ≤ 0.01 | *p* ≤ 0.01 | *p* ≤ 0.01 |

SD = standard deviation; *n* = number of subjects; X = mean
