# Supplementary material for: Psychometric Properties of the Brazilian Version of the Sport Anxiety Scale-2
Source: Front Psychol. 2019 Apr 16;10:806. doi: 10.3389/fpsyg.2019.00806 (PMC6477035; doi:10.3389/fpsyg.2019.00806)
Supplement: Supplementary file 3 [file Table_3.docx]

**Supplementary Table S3.** Indicators of internal consistency of the SAS-2 for the Brazilian context.

| **Subscales** | **Items** | **α if the item is excluded** | **Scale variance if the item is excluded** |
| --- | --- | --- | --- |
| Somatic  α = 0.73 | 2  6  10  12  14 | 0.70  0.70  0.70  0.65  0.68 | 4.03  3.67  3.94  3.71  3.78 |
| Worry  α = 0.86 | 3  5  8  9  11 | 0.82  0.84  0.82  0.80  0.87 | 8.26  8.05  7.88  7.63  9.55 |
| Concentration disruption  α = 0.83 | 1  4  7  13  15 | 0.80  0.78  0.80  0.80  0.82 | 3.77  3.62  4.10  4.00  3.80 |
| **Total score (α = 0.88)** | | | |

α = Cronbach's alpha
