# Supplementary material for: Psychometric Properties of the Brazilian Version of the Sport Anxiety Scale-2
Source: Front Psychol. 2019 Apr 16;10:806. doi: 10.3389/fpsyg.2019.00806 (PMC6477035; doi:10.3389/fpsyg.2019.00806)
Supplement: Supplementary file 4 [file Table_4.docx]

**Supplementary Table S4.** Syntax for calculating the SAS-2 factorial score.

TITLE: CFA SAS2 Model 3 Factors [correl errors Items 6 and 12];

DATA: FILE IS Vivi.txt;

FORMAT IS FREE;

VARIABLE:

NAMES ARE id gender age school indgroup amatprof sas1

sas2 sas3 sas4 sas5 sas6 sas7 sas8 sas9 sas10 sas11

sas12 sas13 sas14 sas15 sumSOM sumWO sumCD sumTOTAL;

MISSING IS id gender age school indgroup amatprof sas1

sas2 sas3 sas4 sas5 sas6 sas7 sas8 sas9 sas10 sas11

sas12 sas13 sas14 sas15 sumSOM sumWO sumCD sumTOTAL (999);

USEVARIABLES ARE sas1 sas2 sas3 sas4 sas5 sas6 sas7 sas8

sas9 sas10 sas11 sas12 sas13 sas14 sas15;

CATEGORICAL ARE sas1 sas2 sas3 sas4 sas5 sas6 sas7 sas8

sas9 sas10 sas11 sas12 sas13 sas14 sas15;

MODEL:

SOM BY sas2 sas6 sas10 sas12 sas14;

WO BY sas3 sas5 sas8 sas9 sas11;

CD BY sas1 sas4 sas7 sas13 sas15;

sas6 WITH sas12;

OUTPUT: STDYX MODINDICES(ALL);

PLOT: TYPE is PLOT3;

SAVEDATA:

FILE IS SAS2_Brasil.sav;

SAVE IS FSCORES;
